# Stability of Vitamins B_1_, B_2_, B_6_ and E in a Fortified Military Freeze-Dried Meal During Extended Storage

**DOI:** 10.3390/foods9010039

**Published:** 2020-01-02

**Authors:** Ross Coad, Lan Bui

**Affiliations:** Defence Science and Technology Group, Scottsdale, Tasmania 7260, Australia; lan.bui@dst.defence.gov.au

**Keywords:** storage stability, vitamins, military nutrition, freeze-dried meal

## Abstract

Australian military ration packs contain a variety of processed foods, including some that are fortified with vitamins. In this study, freeze-dried meals, a key component of lightweight patrol ration packs, were fortified at the time of packing by direct addition of a vitamin premix containing vitamins B_1_, B_2_, B_6_ and E. Fortification was at three levels: 50%, 100% and 200% of the recommended vitamin content for military ration packs. Vitamin stability was determined following storage at temperatures of 1 °C, 30 °C and 40 °C for up to 24 months. HPLC methods were used to measure vitamin content; water activity and colour were also determined. Mean 24-month retention rates across all temperatures and fortification levels were 94%, 97%, 86% and 77% for vitamins B_1_, B_2_, B_6_ and E, respectively. Water activity increased with storage temperature, whereas colour changes due to fortification and storage temperature were at the threshold of visual detection. Fortification of freeze-dried meals would be an effective means of improving the quality of light weight military ration packs by addressing shortfalls in vitamin levels.

## 1. Introduction

Freeze-dried foods are valued for their suitability for hiking, camping, space exploration, emergency and survival applications, and in military ration packs. The utility of freeze-dried foods for both military and civilian consumers, stems from their light weight, long shelf life, sensory quality, portability and ease of preparation for consumption. In contrast, preparation of freshly cooked meals in the field is logistically demanding, and canned or flexible retort pouched meals are heavy and typically of lower sensory acceptability. Military ration packs contain a variety of food products consolidated into a single pack to provide soldiers with their daily dietary intake when access to fresh feeding cannot be provided. In Australia, three types of ration pack are used, a general-purpose combat ration pack and a lightweight patrol ration pack both to feed one person for one day, and a group feeder to feed five persons for one day. The main meals in all types of ration pack are similar to casseroles or stews. A distinguishing characteristic of the patrol ration is the use of freeze-dried meals, whereas the other ration packs contain fully-hydrated meals (retort meals) packed in flexible retort pouches. Australian ration packs are required to have a shelf life of not less than two years at 30 °C, constraining the range of suitable ingredients, processing techniques, and packaging materials.

Freeze-dried products have very low water content (moisture <2%, water activity <0.2), and are nitrogen flushed and vacuum packed in a high barrier laminate pouch, providing protection from light, water and oxygen transmission. These product and packaging characteristics make them ideally suitable for use in a military environment, conferring physical robustness and a long shelf life.

The levels of macronutrients, vitamins and minerals that ration packs should contain at the time of supply to Defence are termed ‘recommended nutritional criteria’ (RNC) [1]. The RNC are intended to ensure the nutritional needs of soldiers working at moderate levels of activity are met and includes allowances for degradation of vitamins while the ration packs are resident in the supply chain. Ration packs may be issued and consumed at any time, from the day of entry into the storage and distribution system, through the ensuing two years or more until they are deemed to be beyond their useful life.

An examination of the nutrient content of ration packs revealed that for the lightweight ration pack the levels of most vitamins were low compared to other ration packs, and for some vitamins, such as vitamin B_6_, the RNC were not being met [2]. Furthermore, it is apparent that for certain vitamins, such as vitamin E, great reliance is placed upon a large contribution from one or two components. Consumption of these items is not guaranteed as soldiers discard about 50% of the contents of their ration packs [3,4].

Most ration pack foods are highly processed and inclusion of some fortified items in a ration pack is necessary to ensure nutrients are present in adequate amounts. Retort pouch meals, used in general purpose ration packs, have long been fortified for the purpose of achieving the required levels of vitamins B_1_, B_2_, B_3_ and C, including allowances for losses during storage; fortification with vitamin B_6_ has also been recommended [1,5].

Losses of labile nutrients during storage can reduce ration pack quality to the point where the nutritional needs of the consumers can no longer be met. The extent to which an individual ration pack component retains its nutritional value throughout the anticipated period of use is an important measure of its fitness for purpose, or quality. However, the freeze-dried meals, used in light weight ration packs, are not fortified at all, although they should be well-suited for fortification, as the loss of nutrients from dehydrated foods depends on light, oxygen, and water activity—well-controlled in these products—and storage temperature [6,7].

In earlier work, beta-carotene, vitamin C, and vitamin E were added during the cooking process, prior to freeze drying and packing as individual serves in vacuum sealed laminate pouches [6]. It was found that the vitamins were stable during storage for 24 months at temperatures up to 37 °C, but significant losses occurred prior to freeze drying—almost 90% for vitamin E—and compensation by a large overage would be required.

Due to the magnitude of processing losses and the risk that these losses would be highly variable in the manually controlled cooking process, an alternative approach was sought. In the current study, vitamins were added directly to a fully processed freeze-dried meal that was subsequently placed on storage. Based on previous work, vitamins B_1_, B_2_, B_6_ and E were identified as vitamins of interest for this study [1,2]. The objectives of this study were to evaluate the effectiveness of a direct dosing approach to vitamin fortification of a freeze-dried meal, and the stability of the added vitamins during storage under conditions relevant to a military supply chain.

## 2. Materials and Methods

### 2.1. Selection of a Suitable Product for Fortification

The range of freeze-dried meal varieties available for this study were meat-based—beef, lamb, and tuna—meals that included vegetables and pasta. All types were considered suitable as carriers of added vitamins, but the tuna mornay meal was lightest in colour and more likely to show colour changes due to addition of fortificants and development of browning during storage, therefore it was selected for this study.

### 2.2. Levels of Fortification

Three levels of fortification were used for each vitamin, based on their RNC values [1]. Levels 1, 2 and 3 were set such that a single serve of freeze-dried meal would provide 50%, 100% and 200% of the RNC amount, respectively. The unfortified meal containing native amounts of vitamins only was designated as Level 0. The outcomes of this work will inform the amounts of fortificants required to achieve the desired contributions to daily intakes, whilst compensating for losses during storage.

A vitamin premix was prepared (Steggall Nutrition Pty Ltd., Salisbury, QLD, Australia) so that all vitamins could be weighed simultaneously. Vitamins were in forms permitted under Schedule 17–2 of the Food Standards Code [8]. Maltodextrin was used as a filler to increase the mass of premix required per weighing to convenient amounts and to minimise the impact of weighing errors. The masses of premix used per vial were 50 mg, 100 mg and 200 mg for Levels 1, 2 and 3 respectively. An example of the vitamin content and the premix used for Level 1 is shown in Table 1.

Individual doses of the vitamin premix were weighed using an analytical balance (model XS205 Dual Range, Mettler Toledo, Columbus, OH, USA) directly into labelled vials that were placed in separate trays for each dosage level (1–3). As the premix would be added to each meal pouch by inverting the vial and tapping three times to dislodge premix clinging to the sides, a correction factor for retained premix was determined by pre-weighing empty vials (n = 97) and weighing again after transfer of premix. It was assumed that any effects due to retention of premix were the same for all vitamins and levels.

### 2.3. Sample Preparation

The freeze-dried meals were prepared in the normal manner in our production facility. The tuna mornay meal contained tuna (41%), onions, pasta, cheese, corn, tomato paste, butter, milk solids, mustard, thickener, chicken stock powder, parsley and pepper. The meal was prepared in a steam jacketed kettle as a single cook of approximately 262 kg, spread on freeze drying trays (4.1 kg per tray), placed in a −20 °C blast freezer, held overnight, then transferred to the freeze dryer (Pilot Freeze Drying Plant, Budge-Ellis Staff Cooperative Ltd., Silverwater, NSW, Australia) and freeze-dried as a single load. The freeze-dried product was removed from the freeze dryer, transferred to 200 L drums that were evacuated and back flushed with food grade nitrogen, sealed and held overnight. Single serve portions (110 g) of freeze-dried meals were weighed using a top loading balance (model ICS469, Mettler Toledo, Columbus, OH, USA) and transferred into a two-layer paper bag (bleached outer 35 gsm, bleached grease resistant inner 38 gsm). The pre-weighed dose of vitamin premix was added to the meal in the bag by inverting the vial and tapping three times to dislodge any premix clinging to the sides. The paper bag was then folded over, pressed to the required thickness using a hydraulic press, transferred to a labelled, foil laminate pouch (polyester 12 µm/polyethylene 25 µm/aluminium foil 25 µm/polyethylene 25 µm/linear low density polyethylene 50 µm) and vacuum sealed. Pouches were held overnight to ensure integrity of the seals (pouches with failed seals would lose vacuum and would be discarded), prior to commencing the storage trial on the following day.

Samples were placed on storage at 1 °C, 30 °C and 40 °C for periods of 6, 12 and 24 months. The storage profile covered the shelf life requirement (2 years at 30 °C), and allowed for evaluation of tolerance to harsher conditions (40 °C) and therefore projected performance during extended storage at 30 °C.

### 2.4. Colour Measurements

Colour was measured in-house by spectrophotometer (UltraScanPRO, Hunter Associates Laboratory, Inc., Reston, VA, USA) to determine colour change due to the addition of fortificants and browning due to storage at elevated temperatures. Whole packets (n = 2) of freeze-dried meal were homogenised by processing for 15 s in a food processor, and colour was measured (n = 4) and recorded as L*a*b* and *Y* values.

Colour difference was calculated using the CIE ΔE2000 formula [9]. Differences in colour are not perceptible by the human eye below a certain value, known as the just noticeable difference (JND) threshold [10].

Browning was calculated as Browning Index (BI) using the formula [11]

100 × ((X − 0.31)/0.17), where X = (a* + 1.75*L*)/(5.645*L* + a* − 3.012b*)

### 2.5. Water Activity

Water activity was measured in house (n = 2) using a water activity meter (Labmaster/Partner, Novasina AG, Lachen, Switzerland), calibrated with Novasina SAL-T 11 and SAL-T 33 standards, 11.3% and 32.8% humidity, respectively. Sample preparation was the same as for colour measurement.

### 2.6. Vitamin Analysis

Vitamin analysis was conducted commercially by National Measurement Institute, Melbourne, Australia. The laboratory was instructed to homogenise each packet in its entirety (110 g, dry, i.e., not reconstituted) prior to analysis. To determine initial vitamin content, 12 replicate pouches were analysed (n = 12), and during the storage trial three replicate pouches per storage point (n = 3) were analysed. The vitamin pre-mix was also analysed (n = 3) to confirm the vitamin concentrations present.

Vitamins B_1_ and B_2_ were extracted by acid hydrolysis followed by enzymatic digestion using alpha-amylase. The sample was digested with 0.05M sulphuric acid in a boiling water bath for 30 min, then rapidly cooled and 2% alpha-amylase solution added. The sample solution was then warmed at 55 °C for 1 h, then filtered. Riboflavin was determined in the filtrate by reverse phase HPLC on a C18 column using fluorescence detection. The mobile phase was 88% acetonitrile and 12% 0.05 M sodium acetate buffer. Fluorescence detection used an excitation wavelength of 450 nm and an emission wavelength of 530 nm. Thiamin in the filtrate was oxidised to thiochrome using potassium ferricyanide. Separation on a C18 column used acetonitrile and sodium acetate buffer, followed by fluorescence detection at an excitation wavelength of 368 nm and an emission wavelength of 440 nm. A recovery, duplicate and reference material control were run once per 10 samples.

Vitamin B_6_ compounds were extracted from the sample using acetate buffer at pH 4.5. Acid phosphatase and glyoxilic acid were then used to convert the various B_6_ forms to pyridoxal. The converted extract was then made to volume, filtered and an aliquot reacted with borohydride to reduce pyridoxal to pyridoxol. Determination was by reverse phase HPLC on a C18 Nova-pak column using fluorescence detection. The mobile phase was 0.0005 M hexanesulfonic acid (sodium salt)/0.05 M potassium dihydrogen phosphate in 8% acetonitrile/92% water, pH 2.5. Fluorescence detection used an excitation wavelength of 295 nm and an emission wavelength of 390 nm. A recovery, duplicate and reference material control were run once per 10 samples.

Vitamin E, determined as tocopherol isomers, was performed on 2–3 g of accurately weighed sample, added to a 250 mL flask with 30 mL alcoholic KOH and saponified with constant stirring overnight at room temperature. The saponified sample was cooled and made to volume in a 50 mL volumetric flask. 10 mL of the solution was loaded onto a ‘Chromobond XTR’ solid phase extraction column and eluted with petroleum ether. The petroleum ether extract was then reduced under rotary evaporation and dried under a stream of nitrogen gas. The sample was then made up to a suitable volume in heptane for HPLC analysis, with separation by normal phase HPLC on a 5 μm silica column using an iso-propanol in heptane mobile phase. Fluorescence detection used an excitation wavelength of 292 nm and emission wavelength of 326 nm. Quantitation was made against tocopherol isomer standards whose concentration was determined by absorbance measurements. A recovery, duplicate and reference material control were run once per 10 samples.

### 2.7. Data Analysis

Statistical analysis was performed using Microsoft Excel for Mac (Version 15.33, 2017). Vitamin storage study results were analysed by one-way analysis of variance (ANOVA) followed by Student’s *t*-test to estimate significance of differences, with *p* < 0.05 considered as significant.

## 3. Results and Discussion

### 3.1. Fortification

Results from the initial samples (n = 12) indicated the mean addition of fortificant per pouch was 45 ± 1.6 mg for Level 1, 95 ± 1.6 mg for Level 2, and 195 ± 1.6 mg for Level 3 (after allowance for premix retained in tubes post-addition). Linearity was estimated by plotting measured concentrations of vitamins against the amounts added; coefficients of determination ranged from 0.9988 to 0.9997 and slopes from 0.80 to 1.01. The relatively low slopes of 0.91 for vitamin B_1_ and 0.80 for vitamin B_2_ suggests these vitamins may not have been present in the premix at the required concentrations. However, analysis of the premix determined that it contained 116% and 95% of the required amounts of vitamins B_1_ and B_2_ respectively. The low values for vitamin B_2_ may be due to exposure to light between weighing doses and addition to the freeze-dried meal, combined with normal analytical variation. Further work under improved lighting conditions would aid in clarifying the validity of this suggestion.

Apparent effectiveness of vitamin fortification, measured as the increase over background vitamin levels as a proportion of the target levels of fortification, ranged from 75% for vitamin B_2_ (range 75–80%) at Level 1 to 101% (range 88–101%) for vitamin E at Level 2. A possible reason for the low values for vitamin B_2_ has already been discussed. The percent effective fortification for vitamin B_1_ was 84–91% and for B_6_ it was 94–99%.

These results confirm the effectiveness of addition of fortificant to individual pouches of processed freeze-dried meal, and establishment of a baseline for the measurement of stability of vitamins during storage. In the routine production environment, manually weighing and transferring each dose would be neither practical nor efficient. However, a powder dosing pipette or in-line powder dosing system may be suitable; further work is required to determine the best approach.

### 3.2. Stability of Vitamins During Storage

**Figure 1:**
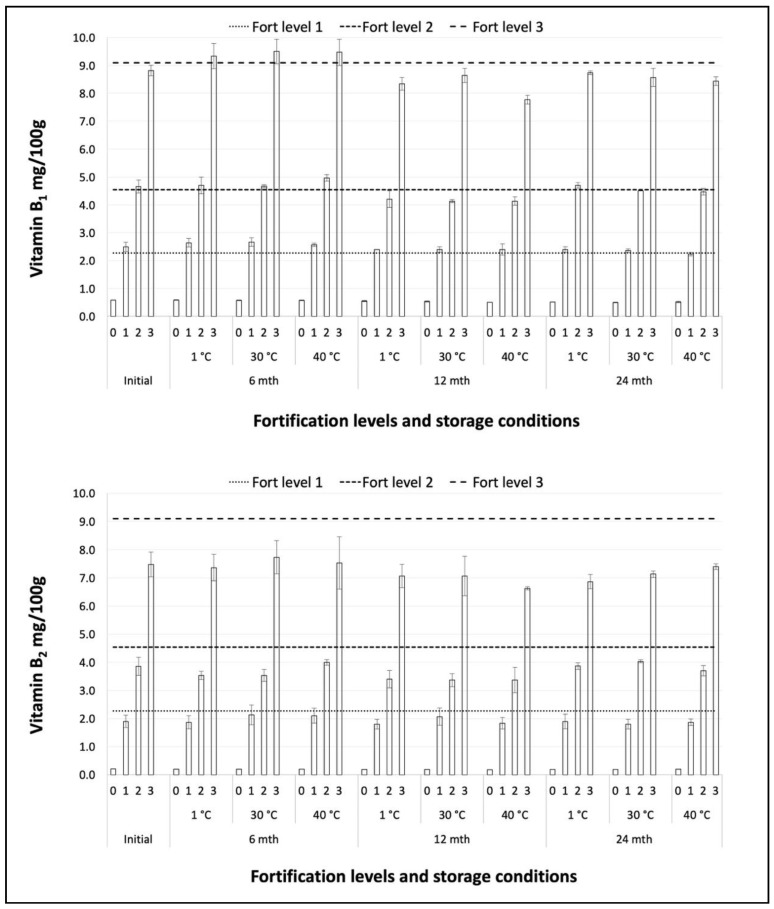
Vitamins B_1_ and B_2_ storage stability at all levels of fortification (0, 1, 2, 3).

**Figure 2:**
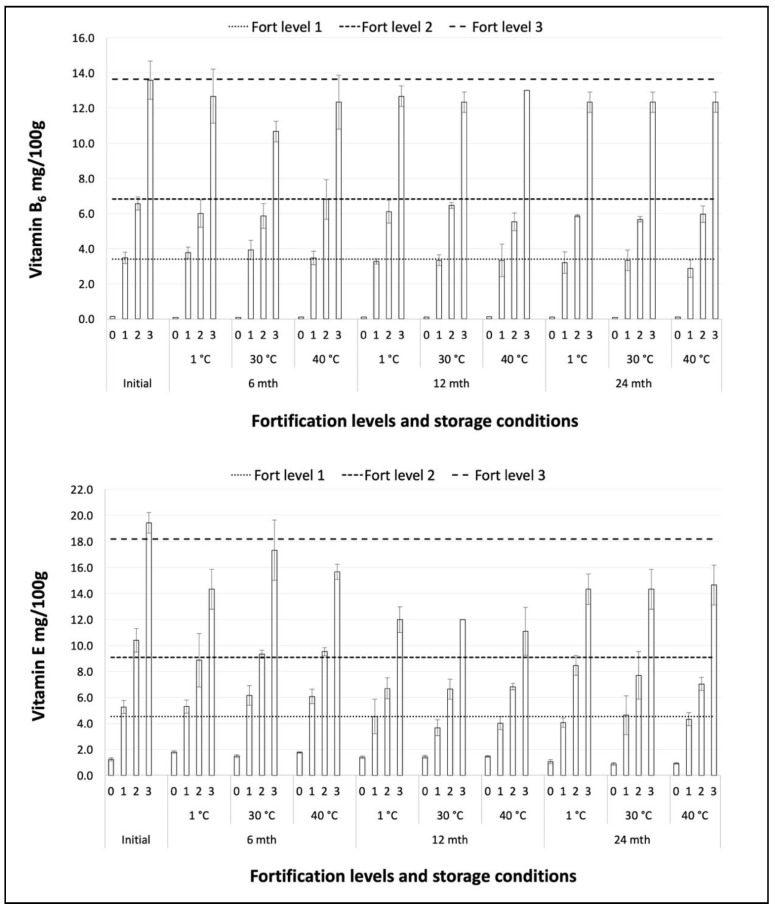
Vitamins B_6_ and E storage stability at all levels of fortification (0, 1, 2, 3).

**Figure 3:**
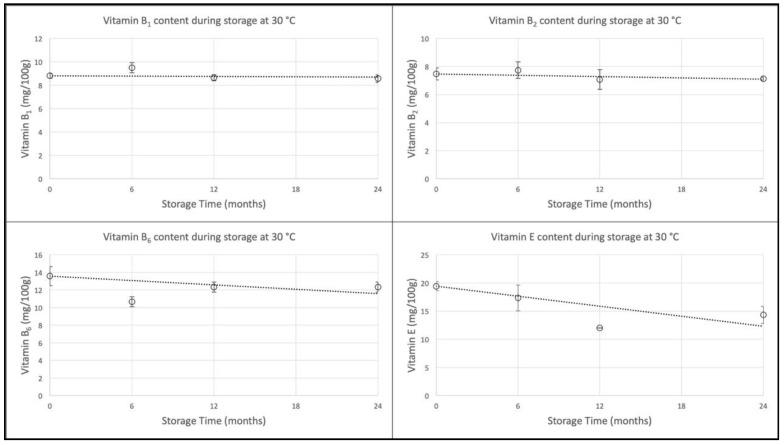
Trend plots forced through the initial values: vitamin fortification Level 3.

The vitamin levels at each stage of the storage trial are shown in Figure 1 and Figure 2. The levels of vitamins B_1_ and B_2_, both native and added, were stable at all temperatures throughout the storage study. Vitamin B_6_ exhibited slight losses during storage, whereas for vitamin E an overall downwards trend was clearer, but storage temperature was not a determinant of vitamin stability. Vitamin E results for all storage temperatures after 12 months of storage were lower than after 24 months of storage. The reason for this is not known, but there may have been an inconsistency in the application of the analytical procedure or conditions leading to relatively low results for the 12-month samples. Both native and added vitamin B_6_ and vitamin E exhibited the same behavior during storage. The levels of all vitamin levels during storage at 30 °C have been plotted and fitted with trend lines forced through the initial values as anchor points (Figure 3).

Vitamin stability during storage was estimated as the degree of retention of the initial level after 24 months of storage, for each level of fortification and each storage temperature (Table 2). Retention was highest for vitamins B_1_ and B_2_ with average percentage retention rates across all levels in the range 93% to 97% for all storage temperatures. The lowest retention rates were for vitamin E at 75%, 77% and 79%, with vitamin B_6_ intermediate at 85%, 86% and 88%, following storage at 1 °C, 30 °C and 40 °C respectively. Mean 24-month retention rates across all temperatures and fortification levels were 94%, 97%, 86% and 77% for vitamins B_1_, B_2_, B_6_ and E, respectively.

Elsewhere, 85% retention has been used as a criterion for acceptability of storage stability [12]. For our purposes, this figure would provide confidence that freeze-dried meals could make a significant contribution to the nutritional value of military ration packs from the day of entry into the storage and distribution system through to the day of consumption, perhaps over two years later. On that basis, our results for vitamins B_1_, B_2_ and B_6_ demonstrate acceptable retention throughout the required shelf life period of two years at 30 °C. Due to the lower retention of vitamin E, further work is needed to understand how its retention can be improved.

Sirmons, Cooper & Douglas evaluated vitamin stability in fortified food products—both freeze-dried and retort pouched—and found that vitamin B_1_ retention in retort pouch products was less than 85% of initial values after storage for 12 months at 35 °C, whereas it was relatively stable in freeze-dried products [12]. Vitamin E retention in both retort pouch and freeze-dried products was greater than 85% after storage for 12 months at 35 °C. In other work, we have observed (based upon in-house results for three studies, where mean retention rates were calculated where initial values were above the level of detection, for vitamin B_1_ (12 meals), vitamin B_2_ (10) and vitamin E (3)) that following storage of wet retort pouch meals for 24 months at 30 °C, vitamin retention rates were 41% for vitamin B_1_, 84% for vitamin B_2_ and 95% for vitamin E. In these studies, vitamin B_6_ levels were initially below the level of detection and retention during storage could not be estimated.

The degradation rate of vitamin E (α-tocopherol) has been found to increase with increasing water activity in the range 0.10–0.65 and increasing temperature in the range 20–37 °C [13]. Esterified forms of vitamin E are reported to be more stable (Ottaway, 2010) [14], therefore we and others have fortified using the acetate form of vitamin E to optimise retention during storage. Vitamin E losses of 50% after 253, 175 and 180 days at 4 °C, 20 °C and 30 °C respectively were observed from apple slices fortified with vitamin E acetate using a vacuum impregnation method [15].

### 3.3. Water Activity During Storage

The mean water activity of samples held at 1 °C for 12 and 24 months was 0.10 ± 0.01 and 0.10 ± 0.02 respectively. This is lower than ideal, as the rate of lipid oxidation is at a minimum at around 0.2–0.4 [16,17]. Drying foods to very low moisture levels has long been recognised as contributing to oxidation and rancidification [18]. However, for vitamin retention, the very low water activity is likely to be beneficial, as the rate of quality loss in food systems begins to increase above water activity 0.2–0.3 for most chemical reactions [16].

There was a trend for water activity to increase with storage temperature, likely due to the release of water in the initial stages of non-enzymatic browning reactions taking place during storage [19]. The mean water activities of samples stored for 12 and 24 months at 40 °C (0.11 ± 0.01 and 0.12 ± 0.02, respectively) were slightly higher compared to samples stored at 1 °C.

### 3.4. Colour Changes During Storage

The colour changes that took place during storage have been summarised in Table 3. The colour difference (CIE ΔE2000) and BI results indicate that although there were significant changes in some measurements of colour, the changes were unlikely to be perceptible to the human eye. Certainly, for the regular consumer of this product, a single serve consumed at one sitting would appear no different to another serve at a subsequent sitting.

JND values ranging from 1 to 5.9 have been reported for CIE ΔE2000 colour differences [20,21]. In the current study, CIE ΔE2000 values calculated for samples of the lowest and highest levels of fortification, and lowest and highest storage temperatures, were found to have values at the lower end of the JND range. However, there was a trend for BI to increase with temperature and for the increase to be statistically significant following storage at 40 °C compared to 1 °C.

As we considered the relatively light-coloured tuna-based freeze-dried meal variety least able to mask colour changes, due to addition of fortificants and development of browning during storage, the study results should be readily extendable to darker coloured freeze-dried meal varieties.

## 4. Conclusions

The work reported here demonstrates that vitamins can be added to individual pouches of freeze-dried meals with sufficient precision to provide confidence in the levels of fortification obtained. Mean 24-month retention rates across all temperatures and fortification levels were 94%, 97%, 86% and 77% for vitamins B_1_, B_2_, B_6_ and E, respectively. Colour changes due to addition of fortificant and development of browning during storage were at or below the threshold for visual detection. The results support the fortification of freeze-dried meals as an effective means of improving the quality of Australian light weight military ration packs by addressing shortfalls in vitamin levels, including with vitamin B_6_, which has not been used previously in the fortification of Australian ration packs.

## Figures and Tables

**Table 1:** Composition of vitamin premix for Level 1 fortification.

Ingredient	Form	Vitamin Per Dose (mg)	Potency	Premix (Vitamin and Filler) Per Dose (mg)
B_1_	Thiamin hydrochloride	2.50	0.7474	3.34
B_2_	Riboflavin	2.50	1.00	2.50
B_6_	Pyridoxine hydrochloride	3.75	0.823	4.56
E	DL α-tocopherol acetate 50%	5.00	0.50	10.00
Filler	Maltodextrin	-	-	29.60
Total				50.00

**Table 2:** Vitamin retention after 24 months of storage.

Temperature	Fortification Level	Vitamin Retention (%)			
		B_1_	B_2_	B_6_	E
1 °C	0	89 ± 2.5 ^1^	93 ± 3.3 ^1^	78 ± 5.6 ^1^	85 ± 17 ^1^
	1	96 ± 7.9	100 ± 18	92 ± 21	77 ± 13 ^1^
	2	101 ± 5.5	100 ± 8.9	89 ± 5.7 ^1^	81 ± 12 ^1^
	3	99 ± 2.3	92 ± 6.9 ^1^	91 ± 9.2	74 ± 9.0 ^1^
30 °C	0	86 ± 2.8 ^1^	93 ± 3.3 ^1^	69 ± 8.2 ^1^	72 ± 14 ^1^
	1	95 ± 7.2	95 ± 15	96 ± 20	88 ± 34
	2	97 ± 5.1	105 ± 8.5	86 ± 6.2 ^1^	74 ± 25 ^1^
	3	97 ± 4.3	95 ± 6.1	91 ± 9.2	74 ± 11 ^1^
40 °C	0	89 ± 5.4 ^1^	96 ± 4.4	76 ± 7.8 ^1^	75 ± 11 ^1^
	1	90 ± 7.2 ^1^	98 ± 13	82 ± 20 ^1^	82 ± 15 ^1^
	2	96 ± 5.7	96 ± 9.6	91 ± 9.7 ^1^	68 ± 11 ^1^
	3	96 ± 2.8 ^1^	99 ± 6.0	91 ± 9.2	76 ± 11 ^1^

^1^ Post-hoc Student’s *t*-test shows significant difference (*p* < 0.05) in vitamin content between initial and 24-month samples.

**Table 3:** Colour results after 24 months of storage.

Fortification Level	Storage Temperature	L* Mean (SD)	a* Mean (SD)	b* Mean (SD)	Y Mean (SD)	BI ^1^ Mean (SD)	CIE ^2^ ΔE2000
0	1 °C	66.3 (0.5)	11.5 (0.4)	26.6 (0.4)	35.7 (0.7)	63.0 (1.8)	
0	30 °C	66.6 (0.6)	11.3 (0.2)	27.7 (0.4)	36.1 (0.7)	65.3 (2.2)	0.7
0	40 °C	66.4 (0.8)	11.7 (0.5)	29.3 (0.5)	35.9 (1.1)	70.0 (3.2)	1.3
1	1 °C	66.4 (0.4)	11.5 (0.4)	26.6 (0.4)	35.9 (0.5)	62.8 (1.4) ^a^	
1	30 °C	67.0 (0.2)	11.5 (0.3)	28.0 (0.3)	36.6 (0.3)	65.7 (0.6) ^b^	0.9
1	40 °C	65.6 (0.4)	11.7 (0.1)	28.8 (0.2)	34.8 (0.5)	69.7 (0.3) ^c^	1.2
2	1 °C	66.7 (1.5)	11.2 (0.2)	26.4 (0.2)	36.3 (2.0)	61.9 (1.5) ^a^	
2	30 °C	67.2 (0.4)	11.1 (0.4)	27.5 (0.4)	36.9 (0.5)	63.8 (1.7) ^a,b^	0.7
2	40 °C	65.6 (0.2)	11.9 (0.1)	28.4 (0.3)	34.8 (0.2)	69.0 (0.7) ^c^	1.3
3	1 °C	67.1 (0.3)	10.7 (0.2)	26.5 (0.2)	36.8 (0.4)	60.9 (0.7) ^a^	0.8 ^3^
3	30 °C	66.2 (0.1)	11.5 (0.1)	28.1 (0.3)	35.6 (0.2)	66.9 (0.3) ^b^	1.1
3	40 °C	65.8 (1.0)	11.3 (0.4)	28.7 (0.3)	35.0 (1.2)	68.8 (1.8) ^c^	1.4

^1^ Browning Index (BI); at each fortification level results with different superscripted letters are significantly different (*p* < 0.05). ^2^ Unless otherwise indicated, at each fortification level the colour differences for 30 °C and 40 °C samples are relative to the 1 °C control sample. ^3^ Colour difference (CIE ΔE2000) has been calculated relative to the fortification level 0, 1 °C control sample.

## References

[1] Forbes-Ewan C. (2009). Australian Defence Force Nutritional Requirements in the 21st Century (Version 1).

[2] Bui L., McLaughlin T., Coad R. (2014). Compliance of 2012/13 Combat Ration Packs to the Recommended Nutritional Criteria.

[3] Carins J.E., Tennant M.L. (2011). Influences on the Consumption of Australian Ration Packs: Review of a Contextual Model and Application to Australian Defence Force Data.

[4] McLaughlin T., De Diana J., Bulmer S., Pike A. (2018). Comparative Field Evaluation of the In-Service and Prototype Modular Mission Adaptive Combat Ration Packs.

[5] McLaughlin T., Bui L., Coad R. (2017). Compliance of 2012/13 Combat Ration Packs to the Military Recommended Dietary Intakes.

[6] Bui L., Coad R. (2011). Suitability of a Freeze Dried Product as a Vehicle for Vitamin Fortification of Military Ration Packs: A Preliminary Study.

[7] Rahman M.S., Labuza T.P., Rahman M.S. (2007). Water activity and food preservation. Handbook of Food Preservation.

[8] Food Standards Australia New Zealand (FSANZ) (2017). Australia New Zealand Food Standards Code—Schedule 17 —Vitamins and Minerals.

[9] Sharma G., Wu W., Dalal E.N. (2004). The CIEDE2000 Color-Difference Formula. Supplemental Test Data and Excel and Matlab Implementations of the CIEDE2000 Color Difference Formula. http://www.ece.rochester.edu/~gsharma/ciede2000/.

[10] Mokrzycki WSTatol M. (2011). Colour difference δE-A survey. Mach. Graph. Vis..

[11] Maskan M. (2001). Kinetics of colour change of kiwifruits during hot air and microwave drying. J. Food Eng..

[12] Sirmons T., Cooper M., Douglas G. (2016). Food fortification stability study. Space Life & Physical Sciences Research & Applications Division Task Book.

[13] Widicus W., Kirk J., Gregory J. (1980). Storage stability of α-tocopherol in a dehydrated model food system containing no fat. J. Food Sci..

[14] Ottaway P.B., Skibsted L.H., Risbo J., Andersen M.L. (2010). Stability of vitamins during food processing and storage. Chemical Deterioration and Physical Instability of Food and Beverages.

[15] Cortés R.M., Chiralt B.A., Suarez M.H. (2009). Influence of storage conditions on freeze-dried apple fortified with vitamin E. Vitae.

[16] Labuza T.P. (1984). Moisture Sorptions: Practical Aspects of Isotherm Measurement and Use.

[17] Troller J.A., Hardman T.M. (1989). Water activity and food quality. Water and Food Quality.

[18] Salwin H. (1959). Defining minimum moisture contents for dehydrated foods. Food Technol..

[19] Hodge J.E. (1953). Dehydrated Foods: Chemistry of browning reactions in model systems. J. Agric. Food Chem..

[20] Azimi M., Boitard R., Nasiopoulos P., Pourazad M.T. Visual Color Difference Evaluation of Standard Color Pixel Representations for High Dynamic Range Video Compression. Proceedings of the 25th European Signal Processing Conference (EUSIPCO).

[21] Larrain R.E., Schaefer D.M., Reed J.D. (2008). Use of digital images to estimate CIE color coordinates of beef. Food Res. Int..

